# The role of mesenchymal stem cells in hematopoietic stem cell transplantation: prevention and treatment of graft-versus-host disease

**DOI:** 10.1186/s13287-019-1287-9

**Published:** 2019-06-21

**Authors:** Lu Zhao, Shanquan Chen, Panxin Yang, Hongcui Cao, Lanjuan Li

**Affiliations:** 1 State Key Laboratory for Diagnosis and Treatment of Infectious Diseases, the First Affiliated Hospital, College of Medicine, Zhejiang University, 79 Qingchun Rd., Hangzhou City, 310003 China; 2 Collaborative Innovation Center for Diagnosis and Treatment of Infectious Diseases, 79 Qingchun Rd., Hangzhou City, 310003 China; 3 The School of Clinical Medicine, University of Cambridge, Cambridgeshire, UK

**Keywords:** Mesenchymal stem cells, Stem cell transplantation, Graft-versus-host disease, Meta-analysis

## Abstract

**Background:**

The use and effectiveness of hematopoietic stem cell transplantation (HSCT) are limited by lethal complications, i.e., acute and chronic graft-versus-host disease (aGVHD and cGVHD, respectively), in which immune cells from the donor attack healthy recipient tissues. GVHD presents both prophylactic and therapeutic challenges, and overall survival is poor. Mesenchymal stem cells (MSCs) show considerable promise in the treatment of GVHD because of their potential immunomodulatory activity. Multiple studies have been performed to explore the possible benefit of MSCs in GVHD, but the results of these studies are sometimes conflicting. Therefore, we performed a systematic review and meta-analysis to estimate the effect of MSC infusion on GVHD treatment and prevention.

**Methods:**

We systematically searched the MEDLINE (PubMed), Cochrane Library, EMBASE, ClinicalTrials.gov, and SinoMed CBM databases to identify studies published before February 2018 involving patients with hematologic malignancies undergoing HSCT and receiving MSC-based or conventional therapy. We included studies if they reported on the outcomes of interest.

**Results:**

Ultimately, 10 studies were selected from among 413 candidates. According to our meta-analyses, compared with conventional treatment, MSC therapy demonstrated substantial improvements in terms of complete response (CR) and overall survival for cGVHD. However, MSC therapy did not show substantial improvements in terms of engraftment, the incidence of aGVHD, relapse, death, death due to relapse, or death due to infection. Subgroup analyses showed that MSCs derived from the umbilical cord (U-MSCs) and MSC infusion after HSCT substantially improved engraftment and cGVHD incidence, whereas MSCs derived from bone marrow (B-MSCs) and MSC infusion before HSCT shows no improvement. In addition, B-MSCs and MSC infusion before HSCT tend to prolong engraftment time, as well as increase the rates of relapse and death.

**Conclusions:**

MSC infusion can reduce cGVHD but not aGVHD incidence and showed a positive effect in patients who already had aGVHD. For GVHD prevention, the use of U-MSCs and MSC infusion after HSCT were optimal for reducing cGVHD incidence and promoting engraftment, and might help decrease the incidence rate of relapse and death. However, B-MSCs and MSC infusion before HSCT may be harmful to patients and thus require serious consideration. A lack of robust evidence, owing to the small number of studies and small sample sizes, indicates a need for further high-quality clinical trials including large numbers of patients to validate our findings.

**Electronic supplementary material:**

The online version of this article (10.1186/s13287-019-1287-9) contains supplementary material, which is available to authorized users.

## Background

Hematopoietic stem cell transplantation (HSCT) is an intensive therapy used to treat hematologic malignant disorders and genetic diseases. Modern approaches to human leukocyte antigen-haploidentical blood or marrow transplantation [1], and improvements of transplantation outcomes, have led to greater use of HSCTs. The number of HSCT procedures continues to increase, with more than 60,000 performed annually according to the Center for International Blood and Marrow Transplant Research [2]. However, its major lethal complication, graft-versus-host disease (GVHD), which may manifest as acute GVHD (aGVHD) or chronic GVHD (cGVHD), limits the effectiveness of HSCT [3].

GVHD is an immunological disorder in which immune cells from the donor attack healthy recipient tissues, including the gastrointestinal tract, liver, skin, and lungs. GVHD occurs in more than 50% of patients undergoing HSCT [4]. According to the extent of involvement of the affected organs, aGVHD is categorized into four types: I (mild), II (moderate), III (severe), and IV (very severe), while cGVHD is subdivided into limited cGVHD and extensive cGVHD. Given the current trend, the number of transplants from unrelated donors is expected to double within the next 5 years and will substantially increase the number of patients with GVHD. The threat posed by GVHD to patient survival is also gradually increasing.

GVHD presents both prophylactic and therapeutic challenges. Prophylactically, pharmacological manipulation of T cells after transplantation is the most commonly used preventive strategy. Administration of antibodies against T cells in vivo for GVHD prevention has been tested extensively using antithymocyte globulin (ATG) and antilymphocyte globulin (ALG) preparations. Unfortunately, neither drug is optimal for enhancing long-term survival, despite reducing the frequency of GVHD. Therapeutically, a steroid regimen, given their potent antilymphocyte and anti-inflammatory effects, remains the gold standard for treatment of GVHD [5]. However, less than half of patients with GVHD who underwent treatment with steroids achieved complete remission; on the contrary, more patients became steroid-refractory and showed very poor overall survival [6–8]. Despite important advances in the field of HSCT over the past few years, there has been little improvement in the morbidity or mortality of GVHD [9]. Patients with severe GVHD have a dismal estimated long-term survival rates, of 25% (5 years) for grade III and 5% for grade IV [10]. Allogeneic transplantation is becoming an increasingly attractive therapeutic option, thus accelerating the search for novel approaches to GVHD.

A cell-based therapeutic approach, using mesenchymal stem cells (MSCs), has recently shown considerable promise because of their expected immunomodulatory effects [11, 12]. MSCs are plate-adhering, fibroblast-like cells known for their self-renewal capacity and ability to differentiate into multiple mesenchymal cell lineages [13]. In addition to their differentiation potential, MSCs are immunosuppressive. Bartholomew et al. first demonstrated that MSC administration in vivo could prolong skin graft survival [14]. Many subsequent in vitro and in vivo studies have confirmed the immunomodulatory activity of MSCs, by showing that they can inhibit the proliferation and functions of T cells, B cells, dendritic cells, and natural killer cells [15–18]. Studies have also reported that MSCs play an active role in promoting facilitation of HSC engraftment following transplantation, because they are part of the HSC niche, wherein they support hematopoiesis [19, 20]. Owing to the growing understanding of MSCs, they have become an exciting tool for treating prophylaxis and GVHD in the HSCT setting and have been approved for use in clinical trials as immunomodulators [21].

Since Le Blanc et al. first reported complete remission of steroid-resistant aGVHD in a child receiving MSC infusions [22], multiple studies have been performed to explore the possible benefit of MSCs in GVHD. However, results are conflicting as to whether MSC infusion during HSCT is effective in managing GVHD [23]. Canada, New Zealand, and some EU countries have approved Prochymal®, the first MSC drug released to the market, for the treatment of children with steroid-refractory GVHD, while other countries including the USA and China have not. The field of MSC therapy is faced with a paradox regarding the clinical utility of MSCs for GVHD, with opposite clinical outcomes in the USA and Europe [23]. A previous meta-analysis [2] of uncontrolled studies with single-arm design was published in 2016 and showed that MSC treatment had a positive effect on 6-month survival in patients with aGVHD. However, no pooled analysis based on controlled trials has confirmed this report. The efficacy of MSC infusion for GVHD prevention is also controversial, with research variously showing both a significant benefit [24] and no benefit [25]. As evidence accumulates, it is essential to explore whether use of MSCs is favorable for GVHD, which represents the first area of clinical application of MSCs. Therefore, we performed a systematic review and meta-analysis of controlled trials to determine the effect of MSC infusion for both GVHD treatment and prevention.

## Methods

### Search strategy and selection criteria

We searched the Medline (PubMed) [26], Cochrane Library [27], EMBASE [28], ClinicalTrials.gov [29], and SinoMed CBM [30] databases up to February 2018 to identify relevant studies using a combined free text and MeSH heading search strategy (see Additional file 1), with no language or time restrictions. The retrieval strategy was based on the patient–intervention–comparison–outcome (PICO) principle and was enhanced by adding keywords related to GVHD (“graft versus host disease”, “graft vs. host disease” and “GVHD”) and mesenchymal stem cells (“mesenchymal stem cell*”, “mesenchymal stromal cell*”, and “multipotent stromal cell*”). We also checked the reference lists of the retrieved studies for additional relevant studies. The inclusion criteria were (1) randomized controlled trials (RCTs) for GVHD prevention, (2) controlled trials for GVHD treatment, (3) inclusion of patients who underwent HSCT, (4) use of MSC, and (5) availability of treatment outcome parameters [complete response (CR) and overall survival] or prevention-related data [engraftment, aGVHD, cGVHD, relapse, death, death due to relapse, and death due to infection]. Studies were excluded if they were animal-based, review articles, or case reports. When duplicate reports from the same study were identified, the one including more information or a longer follow-up period was selected.

### Data extraction and statistical analysis

For each study, data were extracted by one investigator and reviewed by a second investigator to ensure accuracy. Information on the following was extracted: patients (number, age, sex, disease information), MSCs (number, source, dose, number of infusions, and infusion timing), outcome parameters during the follow-up period, and study information (author, publication year, country, study design, and follow-up period).

A meta-analysis was conducted to evaluate whether the efficacy of MSC-based therapy was greater than that of conventional therapy in terms of GVHD prevention and treatment. Outcome parameters were evaluated by calculating the risk ratio (RR) or standardized mean difference (SMD) with 95% confidence intervals (CIs). The percentage of variability across studies attributable to heterogeneity beyond chance was assessed using the chi-square-based *Q* test (*P* < 0.1 was considered indicative of significance) and *I*^2^ statistic (*I*^2^ > 50% indicated high heterogeneity). A forest plot was used to visualize the RR and 95% CI for each study. A random-effects model was used because it provides a more conservative estimate of the presence of heterogeneity. A sensitivity analysis, with omission of one study at a time, was conducted to assess heterogeneity. Where sufficient studies were available, publication bias was assessed by the Egger test and visualized using Begg funnel plots [31]. Subgroup meta-analyses were conducted based on MSC source [bone marrow (B-MSCs) or umbilical cord (U-MSCs)] and MSC infusion timing (before HSCT or after HSCT), to identify factors related to the therapeutic efficacy of MSCs. All analyses were conducted using R software (version 3.4.0; R Development Core Team, Vienna, Austria).

## Results

### Study selection

**Fig. 1:**
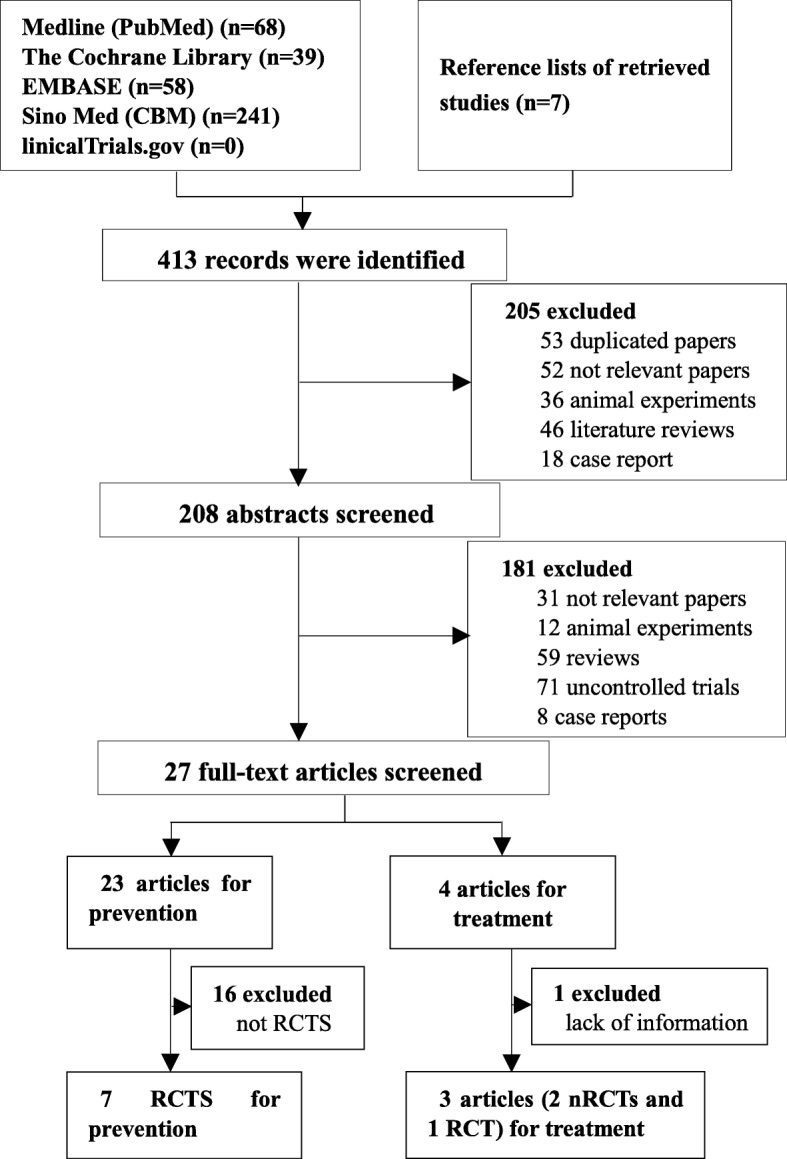
Flowchart of study selection

As shown in Fig. 1, a total of 413 potentially eligible articles were identified by searching the five databases and the reference lists of the retrieved studies. Of these, 53 duplicate articles were excluded. After reading the titles, a further 152 articles were excluded (52 irrelevant papers, 36 animal experiments, 46 reviews and 18 case reports). After reading the abstracts, 181 additional articles were excluded (31 irrelevant papers, 12 animal experiments, 59 reviews, 71 uncontrolled trials, and 8 case reports). Among the remaining 27 articles, 23 concerned prevention and 4 concerned treatment. After assessing the complete texts, 16 of the 23 prevention-related articles were excluded because they were non-randomized controlled trials (nRCTs). The four treatment-related articles included two RCTs and two nRCTs. The two RCTs were meeting abstracts and one [32] of them was excluded because outcome parameters could not be extracted (i.e., survival rate was not reported, and the exact number of cases showing a CR was not available). The remaining three articles were included regardless of whether or not they were RCTs, as controlled trials related to treatment were limited. Thus, seven RCTs [9, 24, 25, 33–36] on prevention and three on treatment (two nRCTs [37, 38] and one RCT [39]) were finally analyzed.

### Characteristics of the included studies

The characteristics of all 10 studies are presented in Tables 1 and 2. The studies were published between 2008 and 2017 and were conducted in China, Russia, Italy, and Sweden. The sample size ranged from 20 to 124 participants with malignant hematological diseases. Seven studies for prevention included a total of 402 patients and three studies for treatment included a total of 103 patients. All patients were treated with HSCT with or without an MSC infusion. The MSCs were B-MSCs or U-MSCs. The dose of infused MSCs ranged from 3.4 × 10^5^ to 7.2 × 10^6^ per kilogram. Infusions were only administered once for prevention, except in one study [9] wherein patients received multiple infusions. Infusions were administered multiple times for treatment, except for one [38] that did not report these data. MSCs were infused before [24, 36] or after [9, 25, 33–35] HSCT to prevent GVHD. For treatment, the median duration of aGVHD before MSC infusion ranged from 8 to 20 days. The follow-up period ranged from 12 to 70 months for prevention and 139 to 1312 days for treatment.

**Table 1:** Character of included studies (prevention)

Study [first author, publish year]	Country	Study design	Patient populations	Sample size (MSC/control)	Average age (MSC/control)	Male (%) (MSC/control)	HLA matching	MSC source	MSC dose	Number of MSC infusions	MSC infusion timing	Maximum follow-up (month)
J Xiang, 2017 [33]	China	RCT	ALL	32/32	5.5/5.2	56.3%/53.1%	6/6	Umbilical cord	1.0 × 10^6^ cells/kg	Once	4 h after HSCT	12
Gao L, 2016 [9]	China	RCT	AML, MDS, ALL,	62/62	18–40 (62.9%)/18–40 (69.4%)	46.8%/48.4%	3/6–5/6	Umbilical cord	3 × 10^7^ cells	No more than 4 doses	Monthly after HSCT	70
Shipounova I N, 2014 [34]	Russia	RCT	Leukemia	39/38	34 (17–63)	NR	6/6	Bone marrow	(0.9–1.65) × 10^6^/kg	Once	19–54 days after HSCT	55
Liu, K, 2011 [25]	China	RCT	ALL, AML, CML, high-risk patients	27/28	30/31.5	74.1%/67.9%	3/6–5/6	Bone marrow	(3–5) × 10^5^ cells/kg	Once	Within 24 h after HSCT	33.5
Ning H, 2008 [24]	China	RCT	AML, CML, MDS, ALL, NHL	10/15	38/37	90.0%/86.7%	6/6	Bone marrow	3.4 × 10^5^ cells/kg	Once	4 h before HSCT	36
Kuzmina L A, 2012 [35]	Russia	RCT	AML,MDS, ALL, CML, CLL	19/18	34/29	42.1%/38.9%	NR	Bone marrow	1.1 × 10^6^ cells/kg	Once	19-54 days (mid 30) after HSCT	32
Wu K H, 2013 [36]	China	RCT	ALL, AML	8/12	9.8/8.5	62.5%/50.0%	4/6–5/6	Umbilical cord	7.2 × 10^6^ cells/kg	Once	4 h before HSCT	27

*ALL* acute lymphoid leukemia, *AML* acute myeloid leukemia, *MDS* myelodysplastic syndrome, *CML* chronic myeloid leukemia, *NHL* non-Hodgkin lymphoma, *CLL* chronic lymphoid leukemia, *NR* not reported

**Table 2:** Character of included studies (treatment)

Study [first author, publish year]	Country	Study design	Primordial therapy	Sample size (MSC/control)	Median age (MSC/control)	Male (%) (MSC/control)	Time of aGVHD diagnosis (mean days after HSCT)	aGVHD grade	MSC source	MSC dose	Number of MSC infusions	Median duration of GVHD prior to enrollment	Maximum follow-up (day)
Zhao K, 2015 [37]	China	nRCT	HSCT	28/19	26/29	67.9%/63.2%	37/33	aGVHD: II-IV	Bone marrow	1 × 10^6^ cells/kg	Once a week (until patients got CR or received 8 doses of MSCs)	17 (11–55)	1312
Szabolcs P, 2010 [39]	Italy	RCT	HSCT	14/14	7/10	50.0%/71.4%	NR	aGVHD: II-IV	Umbilical cord	2 × 10^6^ cells/kg	Once a week for 4 weeks (CR) or 8 weeks (PR)	20/8	139
Remberger, M, 2012 [38]	Sweden	nRCT	HSCT	15/13	57/48	NR	63/56	aGVHD: III-IV	NR	3 × 10^7^ cells	NR	8(0–37)	730

*NR* not reported, *CR* complete response, *PR* partial response

#### Meta-analysis for treatment

Among three articles, a total of 103 patients were suffering from aGVHD, 57 of whom underwent conventional treatment (control group); the remaining 46 patients received additional MSC infusions (MSC group).

### Complete response

**Fig. 2:**
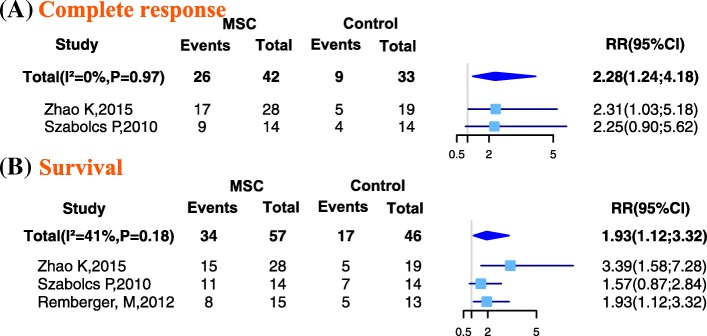
Forest plot of graft-versus-host disease (GVHD) treatment. Compared with the control group, **a** the rate of complete response and **b** the rate of overall survival were significantly higher in the mesenchymal stem cell (MSC) group. Subgroup analyses could not be conducted because of an insufficient number of studies

Two studies reported the number of patients with aGVHD who showed a CR in both MSC and control groups. Compared with the control group, patients in the MSC group had a significantly higher rate of CR (RR = 2.28; 95% CI: 1.24, 4.18; *I*^2^ = 0%, *P* = 0.97; Fig. 2).

### Overall survival

Three studies reported the number of patients with aGVHD who were alive during the follow-up in both the MSC and control groups. Compared with the control group, patients in the MSC group had a significantly higher rate of survival (RR = 1.93; 95% CI 1.12, 3.32; *I*^2^ = 41%, *P* = 0.18; Fig. 2).

#### Meta-analysis for prevention

Among seven RCTs on GVHD prevention, a total of 402 patients with hematologic malignancies underwent HSCT, of whom 205 were undergoing conventional GVHD prevention (control group); the remaining 197 patients received additional MSC infusions (MSC group).

### Engraftment

**Fig. 3:**
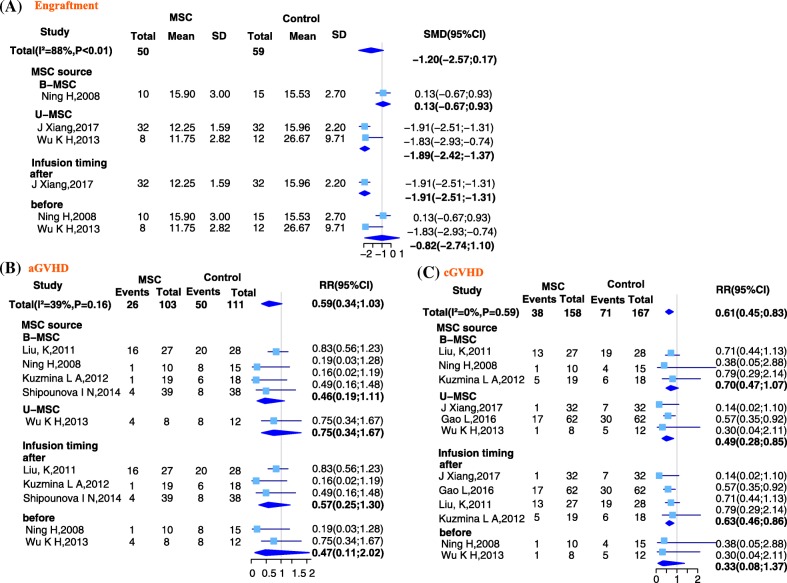
Forest plot of GVHD treatment: whole and subgroup risk estimates of outcome parameters. **a** Forest plot showing the overall risk estimate of engraftment and the effects of MSC source and infusion timing. **b** Forest plot showing the overall risk estimate of acute graft-versus-host disease (aGVHD) and the effects of MSC source and infusion timing. **c** Forest plot showing the overall risk estimate of chronic graft-versus-host disease (cGVHD) and the effects of MSC source and infusion timing

Three studies reported the mean time to neutrophil engraftment (absolute neutrophil count > 0.5 × 10^9^/L) in both the MSC and control groups. Compared with the control group, patients in the MSC group had a shorter time to neutrophil engraftment, but the difference was not statistically significant (SMD = − 1.20; 95% CI − 2.57, 0.17; *I*^2^ = 88%, *P* < 0.01). Significant heterogeneity existed and sensitivity analyses showed that the study of Ning [24] had the largest effect on the heterogeneity. Excluding this study decreased the heterogeneity to a non-significant level (SMD = − 1.89; 95% CI − 2.42, − 1.37; *I*^2^ = 0%, *P* = 0.91). The heterogeneity was likely related to the different sources used of MSCs. According to a subgroup analysis based on the source of MSCs (B-MSCs or U-MSCs), the U-MSC subgroup (SMD = − 1.89; 95% CI − 2.42, − 1.37) showed a significantly shorter time to neutrophil engraftment in the MSC group compared with the control group, whereas the B-MSC subgroup (SMD = 0.13; 95% CI − 0.67, 0.93) showed a longer (but not statistically significant) time to neutrophil engraftment. According to a subgroup analysis based on MSC infusion time (before or after HSCT), the after subgroup (SMD = − 1.91; 95% CI − 2.51, − 1.31) showed a significantly shorter latency to neutrophil engraftment compared with the control group, whereas the before subgroup (SMD = − 0.82; 95% CI − 2.74, 1.10) showed a shorter but not statistically significant time to neutrophil engraftment. These results are shown in Fig. 3.

### Acute GVHD

Five studies reported the number of patients who developed aGVHD within 100 days after HSCT in both the MSC and control groups. Compared with the control group, patients in the MSC group had a lower risk of aGVHD, but the difference was not statistically significant (RR = 0.59; 95% CI 0.34, 1.03; *I*^2^ = 39%, *P* = 0.16). According to a subgroup analysis based on the source of MSC, both the B-MSC (RR = 0.46; 95% CI 0.19, 1.11) and U-MSC (RR = 0.75; 95% CI 0.34, 1.67) subgroups showed a non-significantly lower risk of aGVHD compared with the control group. A subgroup analysis based on MSC infusion time also showed that both the before (RR = 0.47; 95% CI 0.11, 2.02) and after (RR = 0.57; 95% CI 0.25, 1.30) subgroups had a non-significantly lower risk of aGVHD compared with the control group. These results are shown in Fig. 3.

### Chronic GVHD

Six studies reported the number of patients who developed cGVHD in both the MSC and control groups. Compared with the control group, patients in the MSC group had a significantly lower risk of cGVHD (RR = 0.61; 95% CI 0.45, 0.83; *I*^2^ = 0%, *P* = 0.59). According to a subgroup analysis based on the source of MSC, only the U-MSC subgroup (RR = 0.49; 95% CI 0.28, 0.85) showed a significantly lower risk of cGVHD compared with the control group; the B-MSC subgroup (RR = 0.70; 95% CI 0.47, 1.07) showed a non-significantly lower risk of cGVHD. According to a subgroup analysis based on MSC infusion time, only the after subgroup (RR = 0.63; 95% CI 0.46, 0.86) showed a significantly lower risk of cGVHD compared with the control group; the before subgroup (RR = 0.33; 95% CI 0.08, 1.37) showed a non-significantly lower risk of cGVHD. These results are shown in Fig. 3.

### Relapse

**Fig. 4:**
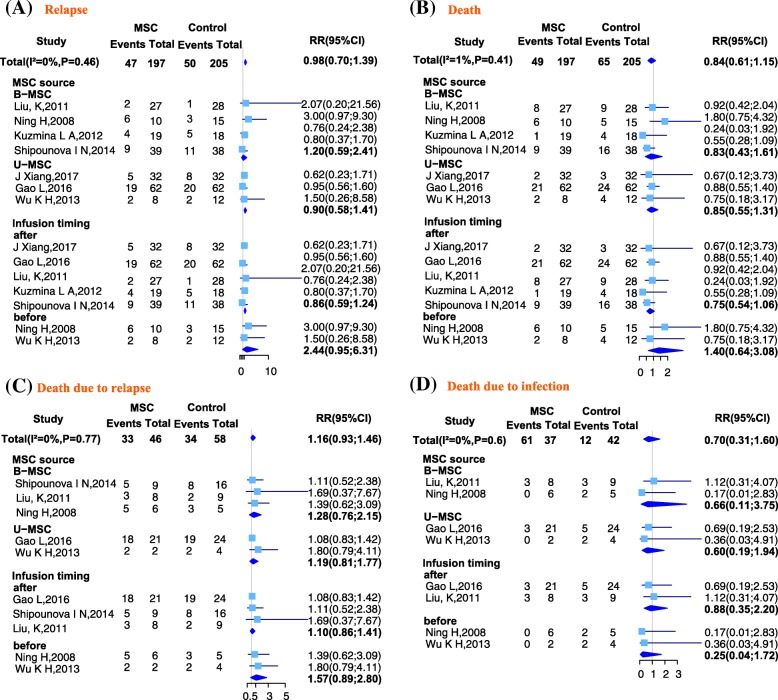
Forest plot of GVHD treatment: whole and subgroup risk estimates of outcome parameters. **a** Forest plot showing the overall risk estimate of relapse and the effects of MSC source and infusion timing. **b** Forest plot showing the overall risk estimate of death and the effects of MSC source and infusion timing. **c** Forest plot showing the overall risk estimate of death due to relapse and the effects of MSC source and infusion timing. **d** Forest plot showing the overall risk estimate of death due to infection and the effects of MSC source and infusion timing

Seven studies reported the number of patients who had relapsed to the original malignant status after HSCT in both the MSC and control group. Compared with the control groups, patients in the MSC group had a lower risk of relapse, but the difference was not statistically significant (RR = 0.98; 95% CI 0.70, 1.39; *I*^2^ = 0%, *P* = 0.46). According to a subgroup analysis based on the source of MSC, the U-MSC subgroup (RR = 0.90; 95% CI 0.581, 1.41) showed a lower (but not statistically significant) risk of relapse compared with the control group, whereas the B-MSC subgroup (RR = 1.20; 95% CI 0.59, 2.41) showed a higher (but not statistically significant) risk of relapse. According to subgroup analysis based on MSC infusion time, the after subgroup (RR = 0.86; 95% CI 0.59, 1.24) showed a lower (but not statistically significant) risk of relapse compared with the control group, whereas the before subgroup (RR = 2.44; 95% CI 0.95, 6.31) showed a higher (but not statistically significant) risk of relapse. These results are shown in Fig. 4.

### Death

Seven studies reported the number of patients who died for any reason after HSCT in both the MSC and control groups. Compared with the control group, patients in the MSC group had a lower risk of mortality, but the difference was not statistically significant (RR = 0.84; 95% CI 0.61, 1.15; *I*^2^ = 1%, *P* = 0.41). According to a subgroup analysis based on the source of MSC, both the B-MSC (RR = 0.83; 95% CI 0.43, 1.61) and U-MSC (RR = 0.85; 95% CI 0.55, 1.31) subgroups showed a non-significant lower risk of mortality compared with the control group. According to subgroup analysis based on MSC infusion time, the after subgroup (RR = 0.75; 95% CI 0.54, 1.06) showed a lower (but not statistically significant) risk of death compared with the control group, whereas the before subgroup (RR = 1.40; 95% CI 0.64, 3.08) showed a higher (but not statistically significant) risk of death. These results are shown in Fig. 4.

### Death due to relapse

Five studies reported the number of patients who died after relapse in both the MSC and control groups. Compared with the control group, patients in the MSC group had a higher risk of death due to relapse, but the difference was not statistically significant (RR = 1.16; 95% CI 0.93, 1.46; *I*^2^ = 0%, *P* = 0.77). According to a subgroup analysis based on the source of MSC, both the B-MSC (RR = 1.28; 95% CI 0.76, 2.15) and U-MSC subgroups (RR = 1.19; 95% CI 0.81, 1.77) showed a higher (but not statistically significant) risk of death due to relapse compared with the control group. A subgroup analysis based on MSC infusion time also showed that both the before (RR = 1.57; 95% CI 0.89, 2.80) and after (RR = 1.10; 95% CI 0.86, 1.41) subgroups had a higher (but not statistically significant) risk of death due to relapse compared with the control group. These results are shown in Fig. 4.

### Death due to infection

Four studies reported the number of patients who died as a result of infection in both the MSC and control groups. Compared with the control group, patients in the MSC group had a lower risk of death due to infection, but the difference was not statistically significant (RR = 0.70; 95% CI 0.31, 1.60; *I*^2^ = 0%, *P* = 0.61). According to a subgroup analysis based on the source of MSC, both the B-MSC (RR = 0.65; 95% CI 0.11, 3.75) and U-MSC (RR = 0.60; 95% CI 0.19, 1.94) subgroups showed a lower (but not statistically significant) risk of death due to infection compared with the control group. Subgroup analysis based on MSC infusion time also showed that both the before (RR = 0.25; 95% CI 0.04, 1.72) and after (RR = 0.88; 95% CI 0.35, 2.20) subgroups had a lower but not statistically significant risk of death caused by infection compared with the control group. These results are shown in Fig. 4.

## Discussion

In this study, we identified, evaluated, and summarized the findings of relevant clinical studies to estimate the efficacy of MSC infusion as a GVHD treatment (one RCT and two nRCTs) and for GVHD prevention (seven RCTs). The findings of our meta-analyses suggest that MSC infusion can reduce cGVHD incidence, but not aGVHD incidence, and has a positive effect on patients who already have aGVHD by increasing the CR rate and prolonging survival.

An increasing number of researchers are noting differences in therapeutic outcomes of MSC treatment among GVHD populations. The use of MSCs for treating aGVHD has received extensive attention, while their use in cases of cGVHD has rarely been reported. In the three trials included in our analysis, only aGVHD was investigated. One possible explanation for this is that the responsiveness of patients with aGVHD to MSCs is superior to that of patients with cGVHD [35]. Since the number of controlled trials investigating MSCs as a treatment for GVHD is limited, we only included three trials. Regardless, our review is the first to evaluate MSC treatment for GVHD in controlled trials and has verified the results of a previous meta-analysis [2] of uncontrolled studies with a single-arm design.

Compared with the treatment of GVHD, controlled trials applying MSCs for GVHD prevention have been widely published. To ensure the reliability of our research, we included only RCTs (*n* = 7) in our analysis. To assess the safety of MSC infusion for GVHD prevention, we performed analyses on the outcomes of engraftment, relapse, and death. Whether or not MSCs increase the incidence of tumor recurrence remains a controversial topic. In fact, MSCs exert bidirectional effects on tumor regulation. On the one hand, MSCs might promote tumor growth and progression, as they can secrete angiogenesis-promoting and suppress immune response-suppressing substances [24]; on the other hand, MSCs may inhibit tumors by activating tumor suppression signaling pathways [40]. Despite some studies showing MSCs increased the risk of tumor relapse [24, 41], our results showed that MSC infusion had no significant effect on the incidence of relapse, death, or time to neutrophil engraftment.

Currently, one of the most successful clinical applications of MSC infusion is involved in HSCT [42]. However, treatment efficacy varies among clinical trials, and several factors might influence this. To identify factors related to the efficacy of MSC infusion, we conducted subgroup meta-analyses for GVHD prevention according to MSC source and MSC infusion timing. MSCs are readily available from a variety of tissues, including bone marrow, umbilical cord blood, adipose tissue, and the placenta. The studies included in this meta-analysis used only B-MSC or U-MSC, probably because these are the MSC types used most commonly in the clinic. Just as the incidence of GVHD can differ according to the use of stem cells derived from bone marrow versus cord blood units [43], the incidence of GVHD also differs according to use of U-MSCs versus B-MSCs. MSC infusion is advantageous for reducing cGVHD incidence, but this was seen only in the U-MSC subgroup and not the B-MSC subgroup in this study. Although MSCs had no overall significant effect on the time to neutrophil engraftment, a significant improvement was seen in the U-MSC subgroup. Regarding relapse, despite no significant difference between the overall meta-analysis and subgroup meta-analysis, use of U-MSCs tended to reduce relapse, whereas use of B-MSCs tended to increase relapse. These findings suggest that B-MSCs are not a good candidate cell type for GVHD prophylaxis in comparison with U-MSCs.

Although adult bone marrow has served as the traditional source of MSCs, fetal-type MSCs, such as U-MSCs, have also proven to be an excellent alternative source [44]. These MSCs can be obtained more easily, proliferate faster in vitro, and show reduced immunogenicity [45–47]. More importantly, compared with adult-type MSCs, fetal-type MSCs have stronger immunosuppressive effects [48]. The reduced immunogenicity and stronger immunosuppressive effects of U-MSCs make them ideal candidates for cell-based therapies, especially for diseases associated with an immune response [48]. The use of U-MSCs for reducing cGVHD incidence and promoting engraftment shows much promise, and further clinical studies involving fetal-type MSCs, such as U-MSCs, in HSCT are urgently needed.

Infusion timing, of which little is known, is another important factor when evaluating the efficacy of MSC-based therapy [49, 50]. Our subgroup analysis of infusion timing revealed that MSC infusion after HSCT had a greater beneficial effect, with significant improvements seen in engraftment and a lower risk of cGVHD incidence occurring only in the after, and not the before, subgroup. In addition, despite a lack of statistical significance, MSC infusion after HSCT tended to reduce the incidence rates of relapse and death, whereas MSC infusion before HSCT tended to increase the incidence rates. The influence of timing of MSC infusion might be related to differences in the immune and inflammation microenvironment in vivo over time [51].

CGVHD is the leading cause of mortality and morbidity after HSCT. Given that our findings showed that MSC infusion decreased cGVHD incidence, its inability to prolong survival seems to be inexplicable and warrants further study. In addition to cGVHD, relapse and infection are the other major causes of death after HSCT [52]. We therefore conducted analyses to determine how MSC infusion affected death due to relapse and death. The results showed that MSC infusion may increase the likelihood of death due to relapse after HSCT. Although the increase was not statistically significant, it remains a concern. Along with the findings showing that the use of B-MSC infusion, and MSC infusion before HSCT, also tended to increase the risk of relapse, MSCs for GVHD prevention failed to enhance survival, which may be related to a higher incidence of relapse and death due to relapse.

There were several limitations to this systematic review. First, we could not precisely estimate the overall treatment efficacy because of the small number of studies and their small sample sizes, especially with respect to GVHD treatment. Although we included both RCTs and nRCTs on GVHD treatment, there were insufficient studies and patients to perform a subgroup meta-analysis. Our findings could be further validated or refined by integrating other data, provided additional relevant literature is published soon. In addition, we were unable to assess publication bias, either statistically or visually, because of the limited number of included studies. Second, possibly because of the small number of studies and their small sample sizes, the statistical power was limited. The possible advantages of U-MSCs over B-MSCs, and of MSC infusion after HSCT versus before HSCT, in terms of decreasing relapse or death, were not statistically demonstrated. Therefore, these results require further validation via additional research. The possible disadvantages of B-MSCs and MSC infusion before HSCT also need to be verified to avoid harming patients. In addition, the possibility that MSC infusion may increase the likelihood of death due to relapse also needs to be further explored. Third, we were unable to assess the effects of some important parameters such as the recipient age, type of MSC donor, type of cancer being treated, preparative therapy before transplantation, and role of HLA mismatching which all could influence the clinical outcomes. In our initial study design, subgroup analyses were to be performed if all seven studies reported the information required and the data could be stratified. Unfortunately however, subgroup analyses based on these variables could not be performed because of an inadequate number of studies or relevant data. These limitations introduced unreliability into our study, but our work should still be interesting to researchers and clinicians devoted to use of MSCs as a safe and effective approach for GVHD.

## Conclusion

In conclusion, the evidence from RCTs and nRCTs suggests that MSCs can play a useful role in HSCT, including by promoting engraftment (U-MSC), preventing GVHD (mainly cGVHD), and ameliorating GVHD (aGVHD). MSC infusion enhanced survival only in the context of treating, and not preventing GVHD. For GVHD prevention, use of U-MSCs and infusion after HSCT were optimal for suppressing cGVHD incidence and promoting engraftment and may decrease the incidence rates of relapse and death. The use of B-MSCs and infusion before HSCT may be harmful to the patient and thus requires serious consideration. In closing, these findings need further confirmation as the limitations imposed on this meta-analysis by the small number of included studies and their small number of patients. Therefore, future studies need to determine the clinical impact of MSC infusion for treating GVHD, and our research needs to be validated via a sufficient number of high-quality clinical trials with large numbers of patients.

## Additional file

Additional file 1: Search strategy. (PDF 12 kb)

## Data Availability

All supporting data are included in the article and its Additional file 1.

## Abbreviations

aGVHD: Acute graft-versus-host disease
ALG: Antilymphocyte globulin
ATG: Antithymocyte globulin
B-MSC: MSC derived from the bone marrow
cGVHD: Chronic graft-versus-host disease
CI: Confidence intervals
CR: Complete response
GVHD: Graft-versus-host disease
HSCT: Hematopoietic stem cell transplantation
MSC: Mesenchymal stem cells
nRCTs: Non-randomized controlled trials
RCT: Randomized controlled trials.
RR: Risk ratio
SMD: Standardized mean difference
U-MSC: MSC derived from the umbilical cord
