# Supplementary material for: The role of mesenchymal stem cells in hematopoietic stem cell transplantation: prevention and treatment of graft-versus-host disease
Source: Stem Cell Res Ther. 2019 Jun 21;10:182. doi: 10.1186/s13287-019-1287-9 (PMC6588914; doi:10.1186/s13287-019-1287-9)
Supplement: Supplementary file 1 — Search strategy. (PDF 12 kb) [file 13287_2019_1287_MOESM1_ESM.pdf]

Additional file 1. Search strategy

Medline (PubMed)

#1 "Mesenchymal Stromal Cells"[Mesh]
#2 mesenchymal stromal cell*
#3 mesenchymal stem cell*
#4 MSC*
#5 #1 or #2 or #3 or #4
#6 graft versus host disease[Mesh]
#7 graft versus host disease
#8 GVHD
#9 #6 or #7 or #8
#10 #5 and #9 Filters: clinical trial, comparative study

Cochrane Library

#1 MeSH descriptor: [Mesenchymal Stromal Cells] explode all trees
#2 "mesenchymal stem cell*":ti,ab,kw (Word variations have been searched)
#3 mesenchymal stromal cell*:ti,ab,kw (Word variations have been searched)
#4 MSC:ti,ab,kw (Word variations have been searched)
#5 #1 or #2 or #3 or #4
#6 "graft versus host disease":ti,ab,kw (Word variations have been searched)
#7 GVHD:ti,ab,kw (Word variations have been searched)
#8 MeSH descriptor: [Graft vs Host Disease] explode all trees
#9 #6 or #7 or #8
#10 #5 and #9

EMBASE

#1 ('graft versus host disease'/exp OR 'graft versus host disease' OR (('graft'/exp OR graft) AND versus AND ('host'/exp OR host) AND ('disease'/exp OR disease)) OR 'gvhd':au) AND [controlled clinical trial]/lim
#2 ('mesenchymal stem cell*' OR (mesenchymal AND ('stem'/exp OR stem) AND cell*) OR 'mesenchymal stromal cell*' OR (mesenchymal AND stromal AND cell*) OR msc) AND [controlled clinical trial]/lim
#3 #1 and #2

SinoMed CBM

(((“mesenchymal stem cell*” [unweighted: extension]) OR “multipotent stromal cell*” [unweighted: extension]) OR “totipotential stem cell" [unweighted: extension]) AND “graft versus host disease” [unweighted: extension]

linicalTrials.gov

“mesenchymal stem cell” and “graft versus host disease” |studies with Results
